# The Prevalence and Characteristics of Frequent Attendees in a Family Medicine Clinic: A Retrospective Cross-Sectional Study at a Primary Health Care Center in Jeddah, Saudi Arabia

**DOI:** 10.7759/cureus.107684

**Published:** 2026-04-24

**Authors:** Lama A Rammal, Reem A Algarni, Anfal A Saber, Faisal S Algaows, Mahmoud A Alzahrani, Raghad A Jar, Mashaer O Alhusaini, Esraa A Alshareef, Amani F Alhakami

**Affiliations:** 1 Department of Family Medicine, Ministry of National Guard Health Affairs, King Abdulaziz Medical City, Jeddah, SAU; 2 College of Medicine, King Saud Bin Abdulaziz University for Health Sciences, Jeddah, SAU; 3 Department of Obstetrics and Gynecology, Ministry of National Guard Health Affairs, King Abdulaziz Medical City, Riyadh, SAU; 4 Department of Internal Medicine, Directorate of Health Affairs, Jeddah, SAU

**Keywords:** family medicine, frequent attenders, healthcare utilization, patient characteristics, primary healthcare, saudi arabia

## Abstract

Background

Frequent attendees in primary healthcare settings pose significant challenges to healthcare delivery systems, impacting resource utilization and service quality. Understanding the characteristics and patterns of frequent attendance is crucial for developing targeted interventions and optimizing healthcare resources.

Objective

To determine the proportion of patients classified as frequent attendees (defined as ≥8 visits per year) among all patients attending the family medicine clinic and to examine their personal (age and gender) and health characteristics (comorbidities, visit purposes, and diagnostic patterns) at the Specialized Polyclinic-Primary Health Care Center (SPC-PHC), National Guard Health Affairs in Jeddah, Saudi Arabia.

Methods

A retrospective observational study was conducted using electronic health record data from patients attending the family medicine clinics at the Specialized Polyclinic-Primary Health Care Center (SPC-PHC), National Guard Health Affairs, in Jeddah, Saudi Arabia, during 2021. Frequent attendees were defined as patients with ≥8 visits within one year. Demographic characteristics, comorbidities, visit purposes, and ICD-10 diagnostic categories were analyzed descriptively.

Results

Frequent attendees represented 6.4% of all unique patients attending the family medicine clinics; however, they accounted for 16.6% of the total visits recorded in the primary healthcare system during the study period. Among frequent attendees, the mean number of visits during the study period was 10.6 (±2.7), while the median number of visits was 10 per patient. Frequent attendees were predominantly female (66.0%), with the majority aged 19-40 years (56.4%). The most common comorbidities were diabetes mellitus (21.3%), dyslipidemia (20.4%), and hypertension (15.4%). New complaints constituted the largest proportion of visits (37.41%), followed by laboratory result follow-ups (15.58%) and chronic issue follow-ups (13.12%). No-show visits accounted for 11.25% of all visits. Endocrine, nutritional, and metabolic diseases (16.65%) were the most prevalent diagnoses, followed by genitourinary (14.96%) and musculoskeletal disorders (14.37%). Associations were observed between visit purposes and patient demographics, with distinct patterns across age groups, gender, and comorbidity status.

Conclusion

The study identified frequent attendance mainly among females aged 19-40 years, with endocrine, genitourinary, and musculoskeletal conditions as leading diagnoses. These findings highlight the need for targeted interventions for frequent attendees, including structured chronic disease management and patient education programs, to improve service efficiency and optimize the use of primary healthcare resources.

## Introduction

Primary health care (PHC) represents the cornerstone of healthcare systems worldwide, serving as the initial point of contact for comprehensive health services. These services encompass integrated curative, preventative, rehabilitative, and palliative care [1]. In the Kingdom of Saudi Arabia, the Ministry of Health (MOH) has significantly expanded its PHC infrastructure [2]. The scale of PHC utilization is substantial [3], averaging 22,473 visits per center [4]. Frequent attendees (FA) represent a significant challenge in primary healthcare delivery. These individuals, characterized by their high utilization rates [5], are typically defined as patients making more than 10 visits annually, while persistent frequent attendees (PFA) maintain this pattern over two consecutive years [6]. The impact of FA extends beyond individual patient care, contributing to increased healthcare costs, heightened provider workload, and diminished satisfaction among both patients and healthcare providers [7]. Understanding this phenomenon is crucial for developing targeted interventions and optimizing resource allocation.

Recent research has identified various factors associated with frequent attendance. A 2021 German systematic review highlighted younger age and unemployment as key predisposing characteristics [7]. Additional studies have revealed correlations with psychological disorders, including anxiety and depression, somatization, and health anxiety [8]. Furthermore, research has consistently shown associations with female gender, musculoskeletal disorders, and mental health conditions [9]. Other contributing factors include perceived poor health status, low quality of life [10], and various sociodemographic characteristics such as marital status, education level, and chronic disease diagnosis [6].

Regional studies provide valuable context, with a 2004 Saudi Arabian study reporting a 27.5% FA prevalence, predominantly among females with chronic diseases and psychosocial problems [5]. Similarly, research from Kuwait comparing 372 FA with 368 non-FA revealed significant differences in visit patterns (16 versus four visits on average) and identified multiple associated factors, including ethnicity, occupation, gender, age, and chronic diseases [11].

Despite the growing recognition of frequent attendance as an important healthcare utilization issue, recent evidence examining frequent attendance patterns in Saudi Arabian primary healthcare settings remains limited, particularly within the National Guard Health Affairs system. Understanding the prevalence and characteristics of frequent attendees in this setting is essential for informing healthcare planning, optimizing resource allocation, and improving primary care service delivery.

Therefore, this study aimed to provide a comprehensive assessment of frequent attendance in a primary healthcare center in Jeddah, Saudi Arabia. The objectives were (1) to determine the proportion of patients classified as frequent attendees (defined as ≥8 visits per year) who visited the Specialized Polyclinic-Primary Health Care Center, National Guard Health Affairs, between January and December 2021, and (2) to analyze their demographic and clinical characteristics, including age, gender, chronic conditions, visit purposes, and ICD-10 diagnostic categories.

## Materials and methods

Study design and area

This retrospective observational study was conducted over a one-year period in 2021 at the Specialized Polyclinic-Primary Health Care Center, National Guard Health Affairs, in Jeddah, Saudi Arabia. During the study period, the center recorded a total of 87,498 visits to general family medicine clinics made by 26,826 unique patients. Although several thresholds have been used in the literature to define frequent attendance in primary care, there is no universally accepted definition. In this study, frequent attendees were operationally defined as patients who made eight or more visits (≥8 visits) to the clinic within one year. This threshold was used to identify patients with relatively high utilization of primary healthcare services within the study setting. Based on this definition, 1995 patients were identified as frequent attendees, accounting for 21,546 visits during the study period.

Study population 

The study population comprised primary health care frequent attendees who made eight or more visits to family medicine clinics during 2021. Patients who attended specialty clinics, including chronic disease clinics, women's health clinics, family planning clinics, and antenatal clinics, were excluded from the study. All eligible patients meeting the inclusion criteria during the study period were included, representing a total population sampling (census).

Data collection

Data were extracted from electronic medical records using a standardized data collection sheet. The electronic medical record system is routinely used for clinical documentation and healthcare management, providing reliable patient data for research purposes. In the electronic health record system, a visit was defined as a scheduled patient encounter recorded in the system, including completed consultations and missed appointments ("no-show" entries). The extracted data were reviewed and cross-checked by the research team to ensure accuracy and completeness prior to analysis. The collected variables included patient demographics (age, gender, and morbidity conditions), number of visits to the general family medicine clinics, the purpose of each visit, and the major diagnosis of each visit. Visit purposes were categorized as new complaints, follow-up of chronic issues, follow-up of laboratory or radiological results, requests for laboratory or radiological investigations, medication refills, specialty referral requests, and missed appointments. The major diagnoses from each visit were coded according to the Tenth Revision of the International Statistical Classification of Diseases and Related Health Problems (ICD-10) [12]. Comorbidities were identified from ICD-10 diagnostic codes recorded in the electronic medical records during the study period (January-December 2021). Major comorbidities were categorized based on ICD-10 groupings, including diabetes mellitus (E10-E14), dyslipidemia (E78), and hypertension (I10-I15).

Statistical analysis

Statistical analyses were performed using SPSS Statistics for Mac, version 29.0 (IBM Inc., Armonk, NY, USA). The final analytic dataset provided for statistical analysis included 1995 patients. Data cleaning resulted in the exclusion of 622 cases: 233 cases from specialty clinics (97 from women's health clinic, 125 from chronic diseases follow-up, and 11 cases for women's health chronic condition follow-up), eight cases due to missing visit frequency data, one case due to missing multiple variables (gender, age, and visit reasons), and two cases with missing age or gender (handled by multiple imputation), and 378 cases due to incomplete or unavailable data. The final analyzed sample included 1373 frequent attendees. Non-frequent attendees were also excluded.

Descriptive statistics were presented as frequencies and percentages for categorical variables, while means, standard deviations, and medians were calculated for continuous variables. The chi-squared test was employed to examine associations between categorical variables among frequent attendees. For analyses of visit purposes, the unit of analysis was individual visits rather than patients. A p-value of less than 0.05 was considered statistically significant for all analyses. The analyses in this study were limited to patients identified as frequent attendees (≥8 visits), and no comparisons with non-frequent attendees were performed. Age groups were categorized differently depending on the analysis. Detailed age categories were used for descriptive analysis, while broader age groups were used in inferential analyses to ensure adequate sample size within each category.

Ethical considerations

The study was conducted after obtaining approval from the Institutional Review Board (IRB) of King Abdullah International Medical Research Center (KAIMRC), Ministry of National Guard Health Affairs, Jeddah, Saudi Arabia (Protocol No. NRJ23J/061/02). Due to the retrospective nature of the study using electronic health records, the requirement for informed consent was waived by the IRB. Patient confidentiality was maintained throughout the study, with no personal identifiers documented during data entry. All data were stored on a password-protected computer, with access restricted to the principal investigator and co-investigators. The findings were reported in accordance with ethical guidelines to ensure data integrity.

## Results

**Figure 1:**
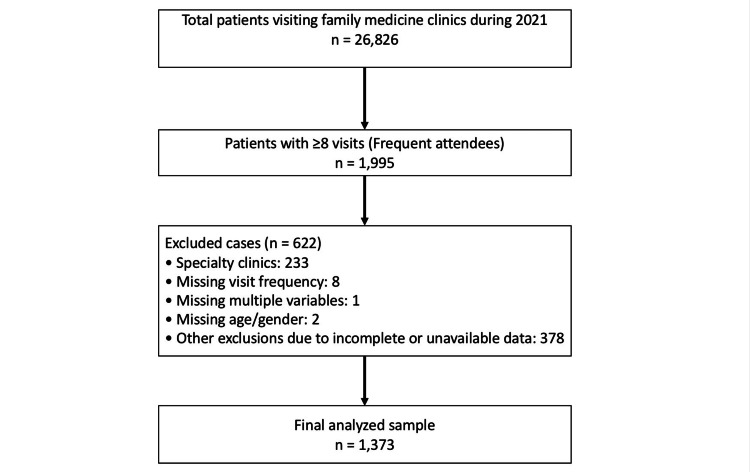
Flow diagram showing the selection of frequent attendees and reasons for exclusion

The results are presented to describe the prevalence of frequent attendees in the study population and to examine their demographic characteristics, comorbidities, visit purposes, and diagnostic patterns. During 2021, a total of 26,826 patients visited the family medicine clinics. Among them, 1995 patients had eight or more visits and were identified as frequent attendees. After applying the exclusion criteria, including specialty clinic visits (n=233), missing visit frequency (n=8), missing multiple variables (n=1), missing age or gender (n=2, handled by multiple imputation), and 378 cases due to incomplete or unavailable data. A total of 1373 patients were included in the final analysis. The selection process of the study population is illustrated in Figure 1.

​​The majority of participants were female (66.0%, n=906) and aged between 19-40 years (56.4%, n=774), followed by those aged 41-60 years (28.5%, n=391). Elderly patients (>60 years) constituted 11.7% (n=160) of the sample, and those ≤18 years represented 3.4% (n=48). Regarding comorbidities, diabetes mellitus was the most prevalent (21.3%), followed by dyslipidemia (20.4%) and hypertension (15.4%). Mental illness and respiratory conditions (asthma/COPD) were present in 6.7% and 6.6% of participants, respectively (Table 1).

**Table 1:** Demographic and clinical characteristics of primary healthcare frequent attendees (N=1373) Frequent attendees were defined as patients with ≥8 visits/year. Other comorbidities include thyroid disorders, inflammatory conditions, and chronic gastrointestinal diseases. COPD - chronic obstructive pulmonary disease

Variable	Groups	n (%)
Age groups	5 years or less	14 (1.0%)
	6-18 years	34 (2.5%)
	19-30 years	328 (23.9%)
	31-40 years	446 (32.5%)
	41-50 years	243 (17.7%)
	51-60 years	148 (10.8%)
	61-70 years	111 (8.1%)
	More than 70 years	49 (3.6%)
Gender	Female	906 (66.0%)
	Male	467 (34.0%)
Co-morbidities		
Diabetes mellitus	Yes	292 (21.3%)
	No	1081 (78.7%)
Dyslipidemia	Yes	280 (20.4%)
	No	1093 (79.6%)
Hypertension	Yes	212 (15.4%)
	No	1161 (84.6)
Mental illness	Yes	92 (6.7%)
	No	1281 (93.3%)
Asthma/COPD	Yes	90 (6.6%)
	No	1283 (93.4%)
Other comorbidities	Yes	101 (7.4%)
	No	1272 (92.6%)

**Figure 2:**
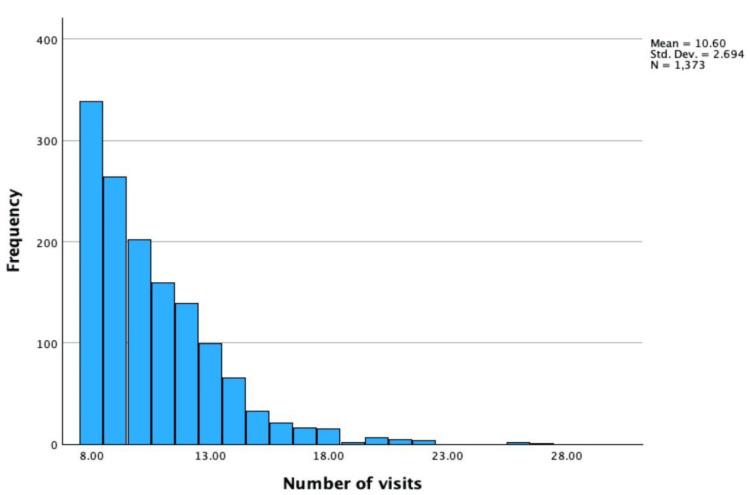
Distribution of primary health care visits among study participants

Among the 26,826 patients who attended the family medicine clinics during 2021, 1373 patients met the definition of frequent attendees, corresponding to a prevalence of frequent attendance of 6.4% among clinic patients. These frequent attendees accounted for 21,546 visits during the study period. Of these, 14,551 visits occurred within the general family medicine clinics and were included in the analytic dataset, representing 16.6% of the total 87,498 visits recorded in the primary health care system during the same period. Further analysis showed that the mean number of visits among frequent attendees was 10.6 (± 2.7) per patient during the study period, with a median of 10 visits per patient (Figure 2).

Analysis of visit purposes revealed that new complaints constituted the largest proportion of visits (37.41%, n=5442), followed by follow-up of laboratory results (15.58%, n=2267) and chronic issues (13.12%, n=1908). Notably, no-show visits accounted for 11.25% of all visits. Regarding primary diagnoses based on ICD-10 classification, endocrine, nutritional, and metabolic diseases were most common (16.65%), followed by diseases of the genitourinary system (14.96%) and musculoskeletal system (14.37%). Mental and behavioral disorders accounted for 1.32% of all diagnoses (Table 2).

**Table 2:** Distribution of visit categories and primary diagnoses based on ICD-10 classification among primary healthcare frequent attendees (N=1373) ICD-10 diagnostic categories were used for disease classification F/U - follow-up

Variable	Groups	n (%)
Purpose of visit	New complaint	5442 (37.41%)
	F/U of chronic issue	1908 (13.12%)
	F/U of lab results	2267 (15.58%)
	F/U of radiology results	489 (3.36%)
	Missed appointment	158 (1.09%)
	Medication refill	1186 (8.15%)
	Request laboratory investigation	941 (6.47%)
	Request radiological investigation	121 (0.83%)
	Request referral	268 (1.84%)
	No show	1636 (11.25%)
	Wrong booking	132 (0.91%)
Diagnosis of the visit	Endocrine, nutritional and metabolic disease	1564 (16.65%)
	Disease of the genitourinary system	1405 (14.96%)
	Disease of the musco-skeletal system and connective tissue	1350 (14.37%)
	Disease of the digestive system	1088 (11.58%)
	Pregnancy, childbirth and the puerperium	779 (8.29%)
	Disease of the respiratory system	669 (7.12%)
	Disease of the skin and subcutaneous tissue	573 (6.10%)
	Infectious and parasite disease	324 (3.45%)
	Factors influencing health status and contact with health services	314 (3.34%)
	Symptoms, signs, and abnormal clinical and laboratory findings, not elsewhere classified	263 (2.80%)
	Disease of the circulatory system	226 (2.41%)
	Disease of the ear and mastoid process	212 (2.26%)
	Disease of the nervous system	170 (1.81%)
	Disease of the eye and adnexa	169 (1.80%)
	Mental and behavioral disorders	124 (1.32%)
	Diseases of the blood and blood-forming organs, and certain disorders involving the immune mechanism	108 (1.15%)
	Neoplasms	34 (0.36%)
	Injury, poisoning, and certain other consequences of external causes	15 (0.16%)
	External causes of morbidity and mortality	4 (0.04%)
	Certain conditions originating in the perinatal period	3 (0.03%)

Analysis of visit purposes across age groups revealed statistically significant variations (p<0.001). The young adult group (19-40 years) showed the highest utilization across most visit categories. This group accounted for 63.2% of new complaints (3393 visits) and 64% of referral requests (169 visits). They also dominated laboratory investigation requests (56%, 521 visits) and radiological investigation requests (64.7%, 77 visits). Medication refills showed a relatively balanced distribution across age groups, with 37.5% (440 visits) among individuals aged 19-40 years, 36.2% (424 visits) in the 41-60 age group, and 24.5% (287 visits) among those over 60 years. Notably, this was the highest proportion of visits for the elderly age group across all visit categories. Follow-up visits for chronic issues also showed a notable pattern, with 52.8% from the 19-40 age group, 29.9% from 41-60, and 14.1% from elderly patients (Table 3).

**Table 3:** Distribution of visit purposes across age groups among primary healthcare frequent attendees (N=1373) Percentages calculated by rows; p-value was calculated using the chi-squared test F/U - follow-up

Purpose of visit	Age groups, N (percentage)				Row total (N)	p-value
	≤18	19-40	41-60	> 60		
New complaint	221 (4.1%)	3393 (63.2%)	1319 (24.6%)	432 (8.1%)	5365	<0.001
F/U of chronic issue	58 (3.1%)	974 (52.8%)	552 (29.9%)	261 (14.1%)	1845	
F/U of lab results	55 (2.5%)	1227 (55.1%)	693 (31.1%)	250 (11.2%)	2225	
F/U of radiology results	15 (3.1%)	217 (44.8%)	171 (35.3%)	81 (16.7%)	484	
Missing appointment	8 (5.2%)	92 (60.1%)	37 (24.2%)	16 (10.5%)	153	
Medication refill	21 (1.8%)	440 (37.5%)	424 (36.2%)	287 (24.5%)	1172	
Request laboratory investigation	22 (2.4%)	521 (56%)	273 (29.4%)	114 (12.3%)	930	
Request radiological investigation	2 (1.7%)	77 (64.7%)	31 (26.1%)	9 (7.6%)	119	
Request referral	8 (3%)	169 (64%)	62 (23.5%)	25 (9.5%)	264	
No-show	45 (2.8%)	988 (62.1%)	398 (25%)	160 (10.1%)	1591	
Wrong booking	5 (3.8%)	75 (57.7%)	41 (31.5%)	9 (6.9%)	130	
Column total (N)	1296	20,898	10,557	4320	14,278	

Detailed gender analysis of visit purposes revealed highly significant differences between male and female patients (p<0.001). Female patients dominated across all visit categories, with particularly striking disparities in certain areas. The most pronounced gender differences were observed in follow-up of radiology results, where females accounted for 72.1% of visits compared to 27.9% for males. This was followed by laboratory investigation requests (females 68.7%, males 31.3%) and follow-up of laboratory results (females 68.5%, males 31.5%). New complaints showed a similar pattern with 67.3% female representation. The pattern persisted but was less pronounced in other categories: chronic issue follow-ups (females 65.8%, males 34.2%) and referral requests (females 63.9%, males 36.1%). The smallest gender disparity was noted in medication refills, where the distribution was more balanced, though still favoring females (59.2% females versus 40.8% males). These findings suggest a consistent pattern of higher healthcare utilization by female patients across all service categories (Table 4).

**Table 4:** Distribution of visit purposes by gender among primary healthcare frequent attendees (N=1373) Percentages calculated by rows; p-value was calculated using the chi-squared test F/U - follow-up

Purpose of visit	Gender		Row total (N)	p-value
	Female n (%)	Male n (%)		
New complaint	3610 (67.3%)	1755 (32.7%)	5,365	<0.001
F/U of chronic issue	1157 (62.7%)	688 (37.3%)	1845	
F/U of lab results	1525 (68.5%)	700 (31.5%)	2225	
F/U of radiology results	349 (72.1%)	135 (27.9%)	484	
Missed appointment	104 (68.0%)	49 (32.0%)	153	
Medication refill	694 (59.2%)	478 (40.8%)	1172	
Request laboratory investigation	639 (68.7%)	291 (31.3%)	930	
Request radiological investigation	99 (83.2%)	20 (16.8%)	119	
Request referral	186 (70.5%)	78 (29.5%)	264	
No show	1052 (66.1%)	539 (33.9%)	1591	
Wrong booking	81 (62.3%)	49 (37.7%)	130	
Column total (N)	9496 (66.5%)	4782 (33.5%)	14,278	

The analysis of visit purposes in relation to comorbidity status revealed significant differences across all categories (p<0.001). Notably, requests for radiological investigations showed the highest proportion of patients with comorbidities (73.9%), followed by missing appointments (67.3%), and new complaints (63.4%). Medication refills demonstrated an inverse pattern, with a higher proportion of patients without comorbidities (63.6%) compared to those with comorbidities (36.4%). Follow-up visits for chronic issues showed a relatively balanced distribution between patients with (49.2%) and without (50.8%) comorbidities. Laboratory-related visits, including both investigation requests and follow-ups, showed similar patterns with slightly higher proportions among patients with comorbidities (56.7% and 56.2%, respectively). These findings suggest that the presence of comorbidities (diabetes mellitus, dyslipidemia, asthma/chronic obstructive pulmonary disease (COPD), mental illness, etc.) influences healthcare utilization patterns, particularly for specific types of services (Table 5).

**Table 5:** Distribution of visit purposes by comorbidity status among primary healthcare frequent attendees (N=1373) Percentages calculated by rows; p-value was calculated using the chi-squared test F/U - follow-up

Purpose of visit	Has a comorbidity		Row total (N)	p-value
	Yes, n (%)	No, n (%)		
New complaint	3400 (63.4%)	1965 (36.6%)	5365	<0.001
F/U of chronic issue	907 (49.2%)	938 (50.8%)	1845	
F/U of lab results	1251 (56.2%)	974 (43.8%)	2225	
F/U of radiology results	270 (55.8%)	214 (44.2%)	484	
Missed appointment	103 (67.3%)	50 (32.7%)	153	
Medication refill	427 (36.4%)	745 (63.6%)	1172	
Request laboratory investigation	527 (56.7%)	403 (43.3%)	930	
Request radiological investigation	88 (73.9%)	31 (26.1%)	119	
Request referral	165 (62.5%)	99 (37.5%)	264	
No show	990 (62.2%)	601 (37.8%)	1591	
Wrong booking	79 (60.8%)	51 (39.2%)	130	
Column total (N)	8207 (57.5%)	6071 (42.5%)	14,278	

## Discussion

The findings of this study highlight important patterns of healthcare utilization among frequent attendees in family medicine clinics. The predominance of younger adults and female patients suggests that demographic factors may influence healthcare-seeking behavior and access to primary care services in this setting. In addition, the presence of chronic conditions such as diabetes, dyslipidemia, and hypertension among frequent attendees indicates that ongoing disease monitoring and long-term management needs may contribute to repeated healthcare utilization. These findings emphasize the importance of strengthening chronic disease management strategies and targeted primary care interventions to optimize healthcare utilization and service efficiency. These findings are consistent with previous studies conducted in primary healthcare settings internationally and within the region, which have also reported higher healthcare utilization among females and patients with chronic conditions.

According to a study that analyzed patient demographics at Riyadh's primary healthcare centers, most attendees were men, and a significant portion were between the ages of 15 and 39 [13]. While these findings are consistent with our results in terms of age groups, gender-related findings contradict our present data, as the majority of our frequent attendees were females. The possible explanation for this frequency could be that women often serve as primary caregivers in families, which may prompt them to seek medical care for themselves and their children or other family members [14]. Women, especially the unemployed group, may have more time to attend healthcare appointments [15]. In addition, certain chronic conditions, such as autoimmune diseases, migraine, and some mental health disorders, are more prevalent among women, which may further contribute to higher healthcare utilization in primary care settings. An association between being female and a higher chance of being a frequent attendee was discovered by a cohort study conducted at an Austrian primary healthcare facility. [16]. These findings align with observations at the studied center, where women were also found to be more frequent attendees compared to men in family medicine clinics. This reflects the role of family medicine clinics as an accessible, first-contact point of care for women in the studied population, highlighting the pivotal role of family physicians in addressing women's health needs comprehensively and promptly.

The present study also evaluated that women consistently utilize healthcare services more than men across most categories. According to a study that involved participants at three community health care centers in the Tshwane Region, South Africa, the majority of participants visiting primary care centers were female. The findings suggest that women were probably attracted to the services because they provided family planning, prenatal care, Pap smears, and vaccinations [17]. Women may also report a high prevalence of chronic conditions such as autoimmune diseases [18] and psychological diseases [19], which require long follow-up and management more actively, further reinforcing our finding.

A longitudinal study on family medicine practices in Slovenia showed that there is no correlation between frequent attendance and age [20]. Nonetheless, a different study conducted in Oman discovered a bivariate correlation between higher age and a greater chance of regular attendance; these results are equivalent to ours, which shows a substantial relationship between age and frequent attendance [5]. The age group of 19-40 years old accounted for the majority of new complaints (63.2%), referral requests (64%), lab requests (56%), and radiology requests (64.7%). Several factors may explain this pattern, including higher exposure to injuries, infections, stress-related conditions, reproductive health needs, and musculoskeletal complaints among younger adults [21].

The distribution of medication refill visits was similar for young and middle-aged individuals. It's interesting to note that young adults had the largest follow-up visits for chronic issues when compared to the elderly. These findings highlight the multifactorial reasons behind the high utilization of family medicine services by the 19-40 age group. These include reproductive health demands, mental health needs, management of chronic diseases, and limitations in access to specialized or structured follow-up services. This underscores the critical role of family medicine clinics as the central hub for comprehensive, accessible, and continuous care for the young and middle-aged population, and calls for targeted health service planning to meet the specific needs of this group while also addressing potential service gaps for older adults.

These results differ from earlier studies, which have reported that a significant proportion of older patients attend larger follow-ups and medical refill appointments [22]. A study conducted among cardiovascular disease patients in Australia showed that patients aged 65-74, 75-84, and ≥85 were significantly more likely to have GP management plans, formal team care, chronic condition reviews, antihypertensive prescriptions, and influenza vaccinations compared to those <65 years of age [22]. Another Canadian cohort study found that older adults aged more than 65 years had higher rates of primary and specialist physician visits and ambulatory care services as compared to younger adults [23]. In contrast, in this study, although older adults (aged ≥60 years) typically have multiple comorbidities, many are already engaged in specialized chronic disease clinics or follow-up in tertiary care facilities, which explains the reduction in their dependence on family medicine services for routine management. Additionally, mobility limitations, transportation barriers, or caregiver availability may also restrict the frequency of healthcare visits among the elderly, even when clinical need exists.

Diabetes mellitus is the most common comorbidity in the present study, and it is closely associated with consistent attendance. Research indicates that diabetes is a prevalent condition among frequent attendees in family clinics in Croatia, where (56.9-62.4%) of individuals with diabetes were frequent attendees, compared to (22.4-24.3%) in the general population [24]. However, the most commonly reported comorbidity was hypertension, which was associated with frequent attendance, according to a case-control study that included 71 frequent attendees and 71 regular attendees from five primary care practices in England [25]. These differences may reflect variations in population demographics, healthcare system organization, access to primary care services, and differences in chronic disease management pathways across healthcare settings. While our study revealed the lowest frequency of individuals with psychiatric difficulties, the case-control study also revealed that frequent attendees had a higher prevalence of mental health issues [25]. This relatively low frequency may also reflect cultural stigma surrounding mental health, underreporting of psychiatric symptoms in primary care records, or referral of patients with mental health conditions to specialized psychiatric services.

Furthermore, our study showed that comorbidity status was associated with differences in the type and frequency of healthcare services used, with a higher proportion of radiological investigation requests among patients with comorbidities (73.9%). In a large community-based study involving 3309 patients, multimorbidity was significantly associated with increased use of healthcare services (p<0.001) [18]. It was further reported that chronic conditions can lead to a marked rise in service utilization, including primary care consultations, hospital outpatient visits, and hospital admissions [18], contributing to substantially increased healthcare costs. These findings are consistent with the pattern observed in our data, where patients with comorbidities demonstrated greater utilization of radiological investigations and other healthcare services. This may reflect the diagnostic complexity and need for disease monitoring, evaluation of complications, and screening for additional conditions commonly associated with comorbidity. According to a retrospective study, patients with multiple long-term conditions, particularly those with mental health disorders, were more likely to miss scheduled appointments even after adjustment for appointment frequency [19]. Similarly, in our study, 67% of patients with comorbid conditions attended their previously missed appointments.

Respiratory system problems were the most common reason for attendance at primary health care institutions, followed by prescription refill services, according to a different survey done in Jeddah City to investigate the most common reasons to visit the primary health care department [26]. In contrast, our study found that endocrine, nutritional, and metabolic diseases accounted for the highest proportion of diagnoses (16.65%), while respiratory symptoms were less commonly reported (7.12%). This may be due to increased patient awareness that many respiratory symptoms are self-limited. Ongoing health education and awareness campaigns led by our center have likely contributed to this understanding, reducing unnecessary visits for minor respiratory complaints and encouraging more focus on chronic conditions like endocrine disorders.

According to a study conducted in the Netherlands, the majority of regular attendees' visits were made to request a physical checkup. Urinary complaints accounted for only 5.3% of frequent attendees, with respiratory diseases and general symptoms being the most common diagnoses [27]. However, 14.96% of frequent attendants in our settings had genitourinary disorders, being the second most common reason for first visit among frequent attendees. The high frequency of genitourinary complaints in family medicine clinics may be explained by easier access compared with specialized services such as family planning or women's health clinics, which often require scheduled appointments. Many female patients prefer the family medicine clinic to seek immediate advice and clarification regarding menstrual disorders, contraception options, complications, and other reproductive health issues. This reflects both the convenience of access and the trusted role of family physicians in addressing sensitive health concerns. Other studies showed that frequent attendants mostly reported acute clinical problems such as enteritis, headaches, and upper respiratory tract infections at polyclinics in Singapore [28].

The findings of this study highlight critical implications for the management of primary health care services in Saudi Arabia, particularly in addressing the needs of frequent attendees. The observed association between frequent attendance and demographic factors such as younger age groups and female gender suggests a need for targeted interventions to enhance access and care delivery for these populations. The study period coincided with the COVID-19 pandemic, which may have influenced healthcare-seeking behavior and contributed to increased primary healthcare visits. Furthermore, the high prevalence of chronic conditions among frequent attendees underscores the importance of integrated chronic disease management programs to reduce the burden on primary care resources [29]. The predominance of visits for new complaints and chronic disease follow-ups indicates the necessity of improving preventive care and patient education to mitigate the need for frequent consultations [30].

This study has several limitations, including the retrospective design, which limits the ability to establish causal relationships between frequent attendance and the associated factors, as the data are based on historical records and may be influenced by unmeasured confounders. In addition, the use of retrospective electronic medical record data may introduce information bias due to potential inaccuracies or incomplete documentation within the clinical records. Additionally, the analysis relied mainly on descriptive statistics and chi-squared tests, and multivariable logistic regression was not performed to adjust for potential confounding factors such as age, sex, and comorbidities. Moreover, the exclusion of patients attending specialty clinics, such as chronic disease, women's health, and antenatal care, may have led to an underrepresentation of individuals with more complex health conditions, affecting the generalizability of the findings. Other unmeasured confounders, such as socioeconomic status, healthcare access, and lifestyle factors, were not accounted for, yet they may have influenced the frequency of visits and health conditions. The study's setting in a single healthcare center in Jeddah further limits its external validity, as the findings may not be fully representative of other primary health care settings or regions with different healthcare systems or demographic characteristics.

## Conclusions

This study examined frequent attendance patterns in a family medicine clinic in Jeddah and identified several important findings. Frequent attendance was most commonly associated with endocrine and metabolic diseases, genitourinary system disorders, and musculoskeletal conditions. The study also identified several common diagnostic categories among frequent attendees, particularly diseases of the genitourinary, respiratory, and digestive systems. Based on these findings, we recommend implementing targeted interventions for managing frequent attendees, including the development of specialized care pathways for patients with chronic conditions, particularly those with endocrine and genitourinary disorders. Additionally, we suggest conducting regular audits of attendance patterns and introducing patient education programs to optimize healthcare utilization. Future research should focus on evaluating the effectiveness of these interventions and exploring the psychological and social factors contributing to frequent attendance patterns in primary healthcare settings.
